# Time’s Up. Descriptive Epidemiology of Multi-Morbidity and Time Spent on Health Related Activity by Older Australians: A Time Use Survey

**DOI:** 10.1371/journal.pone.0059379

**Published:** 2013-04-01

**Authors:** Tanisha Jowsey, Ian S. McRae, Jose M. Valderas, Paul Dugdale, Rebecca Phillips, Robin Bunton, James Gillespie, Michelle Banfield, Lesley Jones, Marjan Kljakovic, Laurann Yen

**Affiliations:** 1 Australian Primary Health Care Research Institute, Australian National University, Canberra, Australian Capital Territory, Australia; 2 Health Services and Policy Research, Department of Primary Care Health Sciences, University of Oxford, Oxford, England; 3 Centre for Health Stewardship, ANU Medical School, Australian National University, Canberra, Australian Capital Territory, Australia; 4 School of Sociology, Australian National University, Canberra, Australian Capital Territory, Australia; 5 Hull York Medical School, University of York, North Yorkshire, England; 6 Menzies Centre for Health Policy and Sydney School of Public Health, Sydney Medical School, University of Sydney, Sydney, New South Wales, Australia; 7 Academic Unit of General Practice, ANU Medical School, Australian National University, Canberra, Australian Capital Territory, Australia; The University of Hong Kong, Hong Kong

## Abstract

Most Western health systems remain single illness orientated despite the growing prevalence of multi-morbidity. Identifying how much time people with multiple chronic conditions spend managing their health will help policy makers and health service providers make decisions about areas of patient need for support. This article presents findings from an Australian study concerning the time spent on health related activity by older adults (aged 50 years and over), most of whom had multiple chronic conditions. A recall questionnaire was developed, piloted, and adjusted. Sampling was undertaken through three bodies; the Lung Foundation Australia (COPD sub-sample), National Diabetes Services Scheme (Diabetes sub-sample) and National Seniors Australia (Seniors sub-sample). Questionnaires were mailed out during 2011 to 10,600 older adults living in Australia. 2540 survey responses were received and analysed. Descriptive analyses were completed to obtain median values for the hours spent on each activity per month. The mean number of chronic conditions was 3.7 in the COPD sub-sample, 3.4 in the Diabetes sub-sample and 2.0 in the NSA sub-sample. The study identified a clear trend of increased time use associated with increased number of chronic conditions. Median monthly time use was 5–16 hours per month overall for our three sub-samples. For respondents in the top decile with five or more chronic conditions the median time use was equivalent to two to three hours per day, and if exercise is included in the calculations, respondents spent from between five and eight hours per day: an amount similar to full-time work. Multi-morbidity imposes considerable time burdens on patients. Ageing is associated with increasing rates of multi-morbidity. Many older adults are facing high demands on their time to manage their health in the face of decreasing energy and mobility. Their time use must be considered in health service delivery and health system reform.

## Introduction

Research on multi-morbidity (defined as the presence of two or more chronic conditions in an individual [1]) has shown an increase in its prevalence over the last decade in Australia and elsewhere [2], [3], [4]. Recent research has focused on tracking patterns of multi-morbidity [2], [3], [4], prescription medication issues [5], [6], [7], the complexity of providing primary care [2], [5], [8], [9], [10], co-ordination [11] and self-management [12], [13].

There is a gap in our knowledge of how people with multi-morbid chronic conditions (multi-morbidity hereafter) use time when undertaking health related activity (HRA). Recently Krueger noted that “Failing to take account of patient time leads us to exaggerate the productivity of the health care sector, and to underestimate the cost of health care” [14]. Drawing on the American Time Use Survey, he estimates that in 2007, Americans spent an average of 1.1 hours each week obtaining healthcare. This time, he argues, is an unseen cost in health care [15], [16], [17]. Other studies have measured the use of time as an unseen cost in health care [15], [16], [17]. Large surveys such as the American and Australian time use surveys provide limited detail about the time people spend on HRA. Current health care models and clinical guidelines can pose unrealistic expectations in terms of the burden of self-management for people with multi-morbidity; who may be prescribed multiple doses of multiple medications each day, and who may also be undertaking several non-pharmacological activities such as exercise, or attending support groups, rehabilitation services or health care services in any given week [18]. Research is needed to address this gap on how people with multi-morbidity spend their time on health care.

### Time to Manage: Priorities in Self-management and HRA

Management of chronic conditions includes self-management as well as interactions with health services, which together comprise HRA. Knowledge of the self-management tasks people perform and their duration has the potential to inform the planning and design of services to support efficient self-management and optimal health outcomes [19], as well as contributing to an understanding of the overall cost to the community of chronic conditions.

Self-management encompasses a range of tasks including managing the medical aspects of the condition (taking medications, testing), maintaining or changing the ways that necessary or meaningful tasks are completed (maintaining a healthy diet, exercising), and coping with the emotions experienced [20], [21]. Performing these tasks is time consuming [15], [19] and is thought to vary between conditions and with severity [15]. Few studies have described the characteristics of people who are likely to spend more or less time managing their health [19]. People with multi-morbidity have management tasks for each condition which can be overwhelming [18], [19], [22].

Patients self-manage because they live with their condition on a daily basis and need to develop strategies to care for themselves [23]. A certain amount of time spent on self-management of chronic condition is inevitable and is necessary [24], [25]. Some activities such as taking prescribed medication cannot be delegated to the system unless a person goes into formal care [12]. Growing evidence supports the effectiveness of self-management to improve health and quality of life outcomes for people with chronic condition [25], [26], and a range of programs are available in Australia to support self management (for example, the Chronic Disease Self Management Program [27] and the Flinders Program [28]). Primary health care services are key spaces in which people learn self-management strategies.

In Australia’s health system clinical guidelines, health policies and care pathways have been developed largely in relation to single illnesses and are focussed on achieving optimum medical outcomes for single conditions. The efficient use of patient time may be taken into account, for example, in cycles of care guidelines for people with diabetes that optimise the time period between various tests (Diabetes Australia 2009 National Evidence Based Guidelines for the Management of Type 2 Diabetes). However, when multiple care pathways are brought into play because a patient has multi-morbidity, the impact on patient time will be quantitatively and perhaps qualitatively different.

The social value of time has been addressed by other research [29], and is not addressed in this study. However we do explore the quantum of time used by people with multi-morbidity to allow some consideration of its impact on their lives. The aim of this study was to quantify the time people with multi-morbidity spend on HRA and its relationship with the number of chronic illnesses using data from The Serious and Continuing Illness Policy and Practice Study (SCIPPS), an Australian study that included research on time use and coordination.

## Methods

The survey built on an earlier qualitative study of 61 patients and 17 informal carers, living with chronic illness in the western suburbs of Sydney and the Australian Capital Territory [30], [31]. The survey was piloted, revised, then mailed to the following groups of older Australians: 5,000 members of National Seniors Australia (NSA - a private body of Australians with 285,000 members aged 50 years and over); 2,500 registrants on the National Diabetes Services Scheme (NDSS - a government funded service which provides subsidies for diabetes materials with 280,000 registrants aged 50 years and over); and 3,100 members who had chronic obstructive pulmonary disease (COPD) of the Lung Foundation Australia (LFA - a private body which supports people with lung conditions).

The sample drawn from NSA members was stratified by State and age (50–64.65–74.75 years and over), with an oversampling of older members to increase the proportions with chronic illness. The sample of registrants aged 50 years or over from the NDSS register, was stratified by State, age (50–59, 60–69,70–79, 80 years and over) and gender with no oversampling as the scheme operates specifically to subsidise costs for persons with diabetes. Samples were selected using simple random sampling within each stratum. All 3,062 members of Lung Foundation Australia with COPD were surveyed. Estimates are weighted by stratum response rates, and analyses undertaken separately for each sub-sample.

The rationale behind this complex sampling framework was that NSA respondents may provide an overview of the problem in the elderly, whereas NDSS targets patients with diabetes, a condition usually associated with co-morbidity [1]. The LFA also provides an illness-specific focus. For ease of reading we use the terms ‘COPD sub-sample’ to reference the LFA sample, and ‘Diabetes sub-sample’ to reference the NDSS sample.

### Ethics Statement

Ethics approval for the survey was obtained from the Australian National University Human Research Ethics Committee (Protocol number: 2010/468) in 2010. All respondents provided informed consent to participate by returning completed questionnaires. As well as taking care over the issues of confidentiality and consent, we were at pains to avoid any additional time burden on the respondents. We therefore tested the length of the questionnaire in the pilot.

### Data Collection

A questionnaire collected data on time use (see Attachment S2). Recall questionnaires were used in this study rather than diaries to limit the burden of the research on the respondents and to encourage response [32], [33], [34]. Time use was defined as the time reportedly spent on any activity in three groups of health-related activities:

1. Activities related to use of medical and allied health services in the previous month; such as making appointments, travelling to health services, waiting in waiting rooms, attending appointments and having medical treatments. These activities are referred to as ‘clinic activities’.
2. Activities related to obtaining information, support or products in the previous month; including attending rehabilitation programs, education programs and support groups, shopping for special foods and looking for/reading health information. These activities are referred to as ‘other activities’.
3. Activities undertaken in domestic spaces on most days (such as time spent on exercising, preparing/consuming prescribed medications, and undertaking tests at home such as blood glucose monitoring). These activities are referred to as ‘home activities’.

The questionnaire also collected data on a range of demographic and other variables including whether people lived in major cities, regional or remote areas, and self-reported use of health services. Australia is a large country where most people live in major cities. The number of chronic conditions was also self-reported, a well-established method for the measurement of multi-morbidity [35]. Respondents were asked ‘Has a doctor ever told you that you had any of the following illnesses?’ This was followed by the list of conditions in Table A in File S1 (see also Attachment S1) and allowed for other conditions to be reported under ‘other’.

### Analysis

Results are presented in terms of hours per month on each grouped activity. As the distribution of time use is highly skewed, results are presented using medians. In order to examine the groups with the highest time use we also examined the time spent by individuals in the top decile of time use. The measure of total time used here excludes exercise unless otherwise stated. While the majority of respondents spent some time on HRA, many people did not spend time on every specific HRA included in the survey (e.g. attending rehabilitation, preparing special foods). When reporting on more detailed components of time use, we therefore report on both the proportion of people undertaking these tasks, and time spent by those undertaking them. Standard errors and confidence intervals were derived using bootstrapping techniques within Stata11 [36]. The Cuzick test for trend was applied for testing trends [37].

## Results

### Survey Response

Overall 2,540 responses were received reflecting an overall response rate of 24.0%, with 427 respondents in the Diabetes sub-sample (16.8% response), 681 in the COPD sub-sample (22.0% response), and 1,432 in the NSA sub-sample (28.4% response). More details of the response rates are shown in Attachment S2. Details of the socio-demographic and chronic condition characteristics of the three sub-samples (weighted for non-response) are shown in Table A in File S1. As expected almost all (94%) of the members of the Diabetes sub-sample reported that they had diabetes and almost all (90%) of the members of the COPD sub-sample reported having COPD. Of the more general NSA population over 40% had hypertension, 35% had arthritis, and over a quarter reported having ever had cancer. Respondents from the COPD and Diabetes sub-samples had on average more co-morbid conditions than the NSA sub-sample (mean number of chronic conditions is 3.7 for the COPD sub-sample, 3.4 for the Diabetes sub-sample and 2.0 for the NSA sub-sample, with COPD/Diabetes difference significant (p = 0.010) and other differences highly significant (p<0.001)). The Diabetes and COPD sub-samples were also prescribed more medications than respondents in the NSA sub-sample (with mean values 4.8, 4.3 and 2.5 respectively, and all differences significant with p<0.001).

### Time Spent on HRA

The time spent on HRA by people in the different demographic and health categories is shown in Table B in File S1 (respondents who spent no time on HRA are included). The reported total median time use per month on HRA excluding exercise was 11.1 hours (95% confidence interval (CI) of 9.3–12.8 hours) for the Diabetes sub-sample, 16.5 (14.7–18.3) hours per month for the COPD sub-sample, and 5.2 (4.7–5.6) hours per month for the NSA sub-sample.

There are few significant differences in time use between age, region, qualifications and income categories although some weak patterns are apparent. The one really clear set of statistically significant time relationships across all three sub-samples is with number of conditions. The number of conditions is related to time use in all sub-samples and is highly significant in all sub-samples (p<0.001 using the Cuzick test for trend [37]). An alternate view of health care complexity is to look at the number of medications taken, particularly since some of the time components relate to medication management. The patterns are broadly in the expected direction for the targeted samples, with the unexpected values for those in small sample categories, and the Cuzick test again shows a very strong relationship (p<0.001) between number of medications and time reported for each sample.

### Components of HRA

As shown in Table C in File S1 almost all respondents spent some time on HRA. People in the COPD sub-sample were most likely to spend time on HRA (97.8% for COPD sub-sample, 95.1% for Diabetes sub-sample and 92.6% for NSA sub-sample). Time use was significantly the highest in the COPD sub-sample (p = 0.017 compared the Diabetes sub-sample and p<0.001 compared with NSA sub-sample). The median time spent on HRA by those who spent time on it was also significantly higher (p<0.001 compared to both other both samples) for the COPD sub-sample (17.5 hours per month) than the Diabetes sub-sample (12.25 hours per month) or the NSA sub-sample (6.0 hours per month).

Table C in File S1 also shows that, excluding exercise, median time spent by all people in the COPD sub-sample (i.e. including those with zero time) and the Diabetes sub-sample on daily home activities was significantly higher (p<0.001) than the time spent on clinical activities or ‘other’ activities. People in the Diabetes sub-sample spent 6.0 hours in the past month on daily activities compared to 1.7 hours on clinic activities. People in the COPD sub-sample spent 7.5 hours on daily activities compared to 3.0 hours on clinic activities. People in the NSA sub-sample spent the same amount of time on daily activities as on clinic activities, but spent less time on the ‘other’ activities. People in the NSA sub-sample were less likely to spend time on all categories than people in the other sub-samples (with differences significant at p<0.001) except the estimated clinic time use for the Diabetes sub-sample and NSA sub-sample were not significantly different). For example, the median time spent on daily activities was only 1.5 hours per month compared to the 6.0 and 7.5 hours per month referred to above.

The reported total median hours (95% CI) on HRA including exercise were 25.8 (22.0–29.5–) hours per month for the Diabetes sub-sample, 31.2 (29.1–33.2) hours for the COPD sub-sample, and 21.7 (20.3–23.0) hours for the NSA sub-sample. Therefore, exercise on average added 14–16 hours per month to median activity, or around half an hour per day. It roughly doubled the estimated median time spent on HRA for the targeted samples and quadrupled it for the NSA sub-sample. Sixteen percent of the NSA sub-sample undertook exercise but no other daily HRA, while there were very few such people in the other samples as nearly all were engaged in some other daily HRA.

### Time Use for the Highest Time Users

To provide an alternate perspective, Table D in File S1 provides the distribution of times for each sub-sample. As can be seen in Table D in File S1, 5.6% of those in the COPD sample reported spending more than 100 hours per month on HRA. Table E in File S1 provides the 90^th^ percentile times showing the total time used (excluding exercise) by the top 10% of the population in each of these categories. The top 10% of time users spent over 51.4 (43.0–59.8) hours per month in the Diabetes sub-sample, over 62.6 (53.5–71.7) hours per month in the COPD sub-sample, and over 34.1 (30.7–37.5) hours per month in the NSA sub-sample on HRA. However, those people with five or more conditions spent 30 to 40 hours per month more than that, with those in the top quintile of the COPD sample who had five or more conditions spending more than 109.5 (85.7–133.3) hours per month which is equivalent to 3.5 hours per day on managing their conditions.

## Discussion

This study has been the first to quantify the time spent on HRA by older Australians with multi-morbidity. The study found that the more chronic illnesses a person had the more time they spent managing their health (especially if they had COPD). Median total time spent in the past month on HRA (excluding exercise) was 16.5 hours for people in the COPD sub-sample, 11.1 hours for people in the Diabetes sub-sample, and 5.2 hours for people in the NSA sub-sample. People in the top 10% of time use from the COPD sub-sample spent 62.6 hours per month or more on HRA, the top 10% of the Diabetes sub-sample spent 51.4 hours per month or more, and the top 10% of the NSA sub-sample spent 34.1 hours per month. Within all sub-samples the time increased with increasing co-morbidity, with estimates of 109.5, 80.1 and 71.5 hours per month for people with five or more conditions in the COPD, Diabetes and NSA sub-samples respectively.

The significantly higher total time for the COPD sub-sample is likely to be due to two factors; 1) that people in this sub-sample had on average more conditions than those in the other sub-samples, and 2) the time demands associated with COPD are higher than many other conditions.

The number of prescribed medications a person takes is also a major and significant determinant of time use, and while numbers of conditions and numbers of medications are clearly correlated they potentially have independent effects on time use. These findings are consistent with our previous qualitative research showing the constraints that multi-morbidity place on the way people spend their time [30].

While the study shows median monthly time use of 5–16 hours per month overall for our three sub-samples, which are not excessive time demands, the demands on those with multi-morbidity become much larger, and people in the top decile of those with five or more conditions face time demands (at the median) equivalent to two to three hours per day. For people with five or more conditions it may be reasonable to assume that exercise is undertaken as part of self-management with a view to optimising health, as many of these people will be restricted by their multiple conditions. Under this assumption, with exercise added the 90^th^ percentile for people with 5 or more conditions is another 30–40 hours per month –110.1 hours, 147.5 hours and 118.5 hours for the Diabetes, COPD and NSA sub-samples respectively. These times are equivalent to between 3.5 and almost five hours per day on average. This means that people with the highest number of conditions in the 90^th^ percentile were spending between 5.5 and eight hours each day on HRA.

The study described the median times spent on HRA either with health services or at home. A gradation of time use for HRA was found with most of the time spent on home activities, followed by time spent on clinic activities and the least time spent on ‘other’ health activities such as shopping for medicines or attending rehabilitation.

While it is not surprising that the study shows that the factors determining time use relate to health it is interesting that other factors do not seem to be material (in particular whether the person lives in a capital city or elsewhere – where travel time costs might have been expected to be important).

### Implications for Self-management Policy and Health Service Delivery

This first study into time use on HRA undertaken by Australians with chronic conditions has shown that illness management occupies considerable time for those with multi-morbidity. These data cannot identify how much of this time is spent on activities which are unnecessary or inefficient (perhaps due to lack of co-ordination). It is clear, however, that clinicians assisting patients with multi-morbidity need to be aware of the time demands made of patients. Options for reducing this demand may include instigating better co-ordination for booking consultations, identifying methods for reducing waiting times, improving support for self-management activities [38], and using straightforward strategies such as pre-packed blister packs for medications or other dose administration aids (DOA). In Australia, pharmacists can dispense medications in DOA, but at an additional cost to the patient that is not presently covered by the Pharmaceutical Benefits Scheme.

On a larger scale, under the current Australian health system reform, strategies are underway to improve team care and care co-ordination [11], [39], [40], [41]. This study provides empirical evidence of the importance of such strategies in terms of decreasing time burdens on people with multi-morbidity. However, as Anstey and colleagues have observed, some approaches to reducing time burdens on both health professionals and patients can have unintentional consequences and the drivers and facilitators of change must be considered carefully. On this matter, Anstey argues that approaches in Australia can learn a lot from those undertaken in other health systems [41].

Finally, for a given level of multi-morbidity, some combinations of illnesses are likely to be associated with higher levels of HRA than others, depending on the concordance or discordance of the illnesses [1]. This issue has not been addressed in this study, and as there are not large differences between time use for particular index illnesses, cluster analysis is likely to be a complex task and will be addressed in a later report.

### Study Limitations

This study had a relatively low response rate, and because of its tripartite structure had relatively small samples in each group. It is possible that people with poor health may have been deterred from responding to the survey and if this is the case then the reported time use may under-estimate the real costs. The Diabetes and COPD sub-samples had lower response rates than the NSA sub-sample. However, as shown in Attachment S1 while response rates varied there were no obvious biases in the non-response, and the usage of the separate samples permitted study of significant numbers of people with diabetes and with COPD.

The study used a recall questionnaire rather than a time use diary to minimize inconvenience to respondents and to extend the period over which the time use could be explored. While there is a known risk of inaccurate recall associated with questionnaires [42] our recent literature review found that they have been utilised in chronic illness research more often than diaries [43].

This study has demonstrated that the time people spend on HRA is substantial and identified a strong gradient in time demands and levels of illness. However, many questions remain unanswered. An important question is how people prioritise their health activities against other activities within the fixed amount of time available each day. Deciding how much time to spend on their health may depend on time scarcity, practical issues and issues of personal prioritisation [44]. Those with multiple conditions or disabilities may also find that they are very slow in performing some of the tasks. Personal prioritisation may be used to make a conscious decision as to whether a social activity will be attended rather than completing a health activity, and healthy choices may yet be made in the social context, thus blurring the lines of time spent on HRA. Russell and colleagues note that “some tasks are more important for certain patients than others” [34∶55] and this study suggests that further more detailed work is required to understand how these decisions are taken.

The study has not captured fluctuations of time use associated with the trajectory of particular conditions. Nor, as also noted above, did the study capture the opportunity costs; the social time costs that are incurred through the chronic illness time costs [14]. To address these problems qualitative research should be undertaken, exploring which options are available to people concerning their time use, which choices people make, and the motivations behind such choices.

### Conclusion

Increasing numbers of chronic conditions are significantly associated with increasing time spent on HRA. On average, people in this study who only had one chronic condition spent between three and 13 hours each month on HRA, depending on the sub-sample. However, people with five or more chronic conditions spent on average between 16 and 27 hours each month on HRA, depending on the sub-sample. For those in the top decile of people with five or more chronic conditions in the COPD sub-sample the time spent on HRA was as high as 110 hours per month. Increasing numbers of prescribed medications is also significantly associated with increasing time spent on HRA. We suggest that in planning future self-management programs, health care services and health policies, considerations be made in terms of patient time use; the costs and benefits to people with multi-morbidity, who may be experiencing significant constraints on their time and changes to the way they use and experience that time.

## Supporting Information

Attachment S1 **Final survey.**
(PDF)

Attachment S2 **Response rates.**
(DOCX)

File S1 **Tables (A–E).**
(DOCX)
