# Supplementary material for: Time’s Up. Descriptive Epidemiology of Multi-Morbidity and Time Spent on Health Related Activity by Older Australians: A Time Use Survey
Source: PLoS One. 2013 Apr 1;8(4):e59379. doi: 10.1371/journal.pone.0059379 (PMC3613388; doi:10.1371/journal.pone.0059379)
Supplement: Attachment S1 — Final survey. (PDF) [file pone.0059379.s001.pdf]

Menzies Centre for Health Policy

THE AUSTRALIAN NATIONAL UNIVERSITY

THE UNIVERSITY OF SYDNEY

How much work is involved in looking after your health?

Thank you for participating in this study. Your answers will provide important information about how much time and effort people spend on looking after their health or the health of someone they care for.

It will take about 20 minutes to complete the questionnaire. You can complete the questionnaire here, using this paper copy, or you can complete it online at the website listed on the covering letter. If you complete this paper copy, could you return it within ***2 weeks*** using the addressed postage paid envelope provided.

Please complete Part A and Part B of the questionnaire. If you look after someone else, please also complete Part C.

Please feel free to ask a friend or a family member to help you with the questionnaire if you want.

How to fill in the questionnaire

- Use a pencil or a blue/black pen
- Do not use a red or felt tip pen
- Do not use liquid paper
- Please put an X in appropriate boxes; do not use ticks.

Some questions ask you to write an answer in a box.

Example: **What type of cancer?** [skin]

How many people altogether live in your household? [0 | 4]

Some questions have a combination of written answers and response boxes. Please ensure you answer each relevant row or part of the question.

Example:

	Yes	How old were you when you were *first* told?
**Do you have cancer?**	[X] 1	[5 | 7]

If you make a mistake in pencil, please erase fully. If in pen, block out the incorrect answer like this: [■] 1 and put an X in the correct box, like this: [X] 2

Part A — Demographics

SECTION 1: PERSONAL AND FAMILY DETAILS

1 What is your year of birth? *Write neatly in the box*

1	9		

2 What is your gender?

- ☐ 1 Male
- ☐ 2 Female

3 Are you of Aboriginal or Torres Strait Islander origin?

- ☐ 1 No
- ☐ 2 Yes, Aboriginal
- ☐ 3 Yes, Torres Strait Islander
- ☐ 4 Yes, both Aboriginal and Torres Strait Islander

4 In which region were you born?

- ☐ 1 Australia/New Zealand
- ☐ 2 UK/Ireland
- ☐ 3 Europe
- ☐ 4 Asia
- ☐ 5 Africa
- ☐ 6 Middle East
- ☐ 7 USA/Canada
- ☐ 8 South America
- ☐ 9 Other

5 Do you speak a language other than English at home?

- ☐ 1 Yes
- ☐ 2 No

6 What best describes your *current* marital status? *Mark one only*

- ☐ 1 Never married
- ☐ 2 Married
- ☐ 3 De facto/Living with a partner
- ☐ 4 Widowed
- ☐ 5 Divorced
- ☐ 6 Separated

7 How many people altogether live in your household?

☐☐

8 How many children under age 15 live in your household?

☐☐

9 What is the postcode of your current residence?

☐☐☐☐

10 What is the highest qualification you have completed? *Mark one only*

- ☐ 1 No school certificate or other qualifications
- ☐ 2 Year 9/Year 10
- ☐ 3 Year 12
- ☐ 4 Trade/apprenticeship (eg hair dresser, chef)
- ☐ 5 Certificate/diploma (eg child care, technical)
- ☐ 6 University degree or higher

11 What is your annual *household* income, before tax, *from all sources*? *Please include any benefits, pensions, superannuation etc. Mark one only*

- ☐ 1 Under $10,000
- ☐ 2 $10,000-$19,999
- ☐ 3 $20,000-$29,999
- ☐ 4 $30,000-$39,999
- ☐ 5 $40,000-$49,999
- ☐ 6 $50,000-$59,999
- ☐ 7 $60,000-$69,999
- ☐ 8 $70,000-$79,999
- ☐ 9 $80,000-$89,999
- ☐ 10 $90,000-$99,999
- ☐ 11 $100,000-$119,999
- ☐ 12 $120,000-$149,999
- ☐ 13 $150,000 or above
- ☐ 14 I prefer not to answer this question

SECTION 2: EMPLOYMENT

12 What *best* describes your current work status? *Mark one only*

- ☐ 1 Full time paid work → GO TO Q15
- ☐ 2 Part time paid work → GO TO Q13
- ☐ 3 Retired → GO TO Q14.1
- ☐ 4 Home duties → GO TO Q15
- ☐ 5 Unemployed and looking for work → GO TO Q15
- ☐ 6 Other (eg student, voluntary worker) *(Specify)* → GO TO Q15

Please check "go to"!!

13 **If you are in part time paid work, what is the *main* reason you choose to work part time?**
Mark one only

- ☐ 1 Health reasons
- ☐ 2 Lifestyle reasons
- ☐ 3 To care for family member or friend
- ☐ 4 Other (Specify) ______
- ☐ 5 Not applicable

➔ GO TO Q15

14.1 **If you are retired, how old were you when you retired?**

☐☐ *years*

14.2 **What was the *main* reason for your retirement?**
Mark one only

- ☐ 1 Reached retirement age
- ☐ 2 Health reasons
- ☐ 3 Lifestyle reasons
- ☐ 4 To care for a family member or friend
- ☐ 5 Other *(Specify)* ______

Part B — Health, coordination and time use

SECTION 1: HEALTH

15 **In general, would you say your health is:**

- ☐ 1 Excellent
- ☐ 2 Very good
- ☐ 3 Good
- ☐ 4 Fair
- ☐ 5 Poor

Cross one box in each group below to indicate which statements best describe your health state TODAY.

16.1 **Mobility**

- ☐ 1 I have no problems with walking about
- ☐ 2 I have some problems with walking about
- ☐ 3 I am confined to bed

16.2 **Self care**

- ☐ 1 I have no problems with self care
- ☐ 2 I have some problems with washing and dressing myself
- ☐ 3 I am unable to wash and dress myself

16.3 **Usual activities** *eg work, study, housework, family or leisure activities*

- ☐ 1 I have no problems performing my usual activities
- ☐ 2 I have some problems performing my usual activities
- ☐ 3 I am unable to perform my usual activities

16.4 **Pain/Discomfort**

- ☐ 1 I have no pain or discomfort
- ☐ 2 I have moderate pain or discomfort
- ☐ 3 I have extreme pain or discomfort

16.5 **Anxiety/Depression**

- ☐ 1 I am not anxious or depressed
- ☐ 2 I am moderately anxious or depressed
- ☐ 3 I am extremely anxious or depressed

16.6 **To help you describe how good or bad your health is, we have drawn a scale on which the best state is 100 and the worst state is 0.**

Place an X in one of the boxes on the scale that indicates how good or bad your health state is TODAY.

Best imaginable

100 ☐
☐
90 ☐
☐
80 ☐
☐
70 ☐
☐
60 ☐
☐
50 ☐
☐
40 ☐
☐
30 ☐
☐
20 ☐
☐
10 ☐
☐
0 ☐

Worst imaginable

17 **Has a doctor ever told you that you have any of the following illnesses?**

	Yes *Mark X for all that apply*	How old were you when you were *first* told?	Were you treated for this illness *in the last 3 months*? *Mark X for all that apply*
Cancer *What type of cancer?* ☐	☐	☐☐	☐
Heart disease *What type of heart disease?* ☐	☐	☐☐	☐
High blood pressure or Hypertension	☐	☐☐	☐
Stroke	☐	☐☐	☐
Diabetes	☐	☐☐	☐
Renal or kidney disease	☐	☐☐	☐
Asthma or Hay Fever	☐	☐☐	☐
Chronic Bronchitis or Emphysema or COPD *(Chronic Obstructive Pulmonary Disease)*	☐	☐☐	☐
Arthritis	☐	☐☐	☐
Osteoporosis	☐	☐☐	☐
Chronic pain, including back pain	☐	☐☐	☐
Depression or Anxiety	☐	☐☐	☐
Other mental health condition	☐	☐☐	☐
Other illness lasting more than 6 months *(Specify)* ☐	☐	☐☐	☐
☐	☐	☐☐	☐

18 ***In the last year*, how many times have you been to an *emergency department* because of one of these illnesses?**

☐☐

19 ***In the last year*, how many times have you had to stay in *hospital* because of one of these illnesses?**

☐☐

20 Do you have private health insurance?

☐ 1 Yes ☐ 2 No

21 Do you keep a record of your test results, medicines or treatment instructions?

	Yes	No
A paper-based record	☐ 1	☐ 2
A computer-based record	☐ 1	☐ 2
A web-based record	☐ 1	☐ 2

22 Do you use the internet to get information about health or illness?

☐ 1 Yes ☐ 2 No

23 Does anyone help you with your daily life activities because of your illness?
Mark X for all that apply

☐ 1 Yes, my partner/spouse
☐ 2 Yes, another family member
☐ 3 Yes, a friend
☐ 4 Yes, a paid helper
☐ 5 No
☐ 6 Not applicable

SECTION 2: SERVICE USE

24 This question asks about the number of health professionals you have received advice or treatment from ***in the last 3 months***.

	How many of the following health professionals have you seen <u>in the last 3 months</u>? eg you may have seen 2 GPs and 1 specialist, or 1 specialist and 1 practice nurse.	How many consultations or treatments (in total) have you had with these health professionals <u>in the last 3 months</u>? eg you may have seen the GPs 3 times and the specialist once. *Mark X for all that apply* Once	2-3 times	4-5 times	6 or more times
General Practitioners (GPs)	☐☐	☐ 1	☐ 2	☐ 3	☐ 4
Specialists	☐☐	☐ 1	☐ 2	☐ 3	☐ 4
Practice nurses	☐☐	☐ 1	☐ 2	☐ 3	☐ 4
Community nurses	☐☐	☐ 1	☐ 2	☐ 3	☐ 4
Pharmacists	☐☐	☐ 1	☐ 2	☐ 3	☐ 4
Allied health professionals *(eg physiotherapist)*	☐☐	☐ 1	☐ 2	☐ 3	☐ 4
Complementary health practitioners *(eg Naturopath)*	☐☐	☐ 1	☐ 2	☐ 3	☐ 4
Mental health workers, psychologists, counsellors	☐☐	☐ 1	☐ 2	☐ 3	☐ 4
Audiologists	☐☐	☐ 1	☐ 2	☐ 3	☐ 4
Optometrists	☐☐	☐ 1	☐ 2	☐ 3	☐ 4
Dentists	☐☐	☐ 1	☐ 2	☐ 3	☐ 4
Other *(Specify)* ________	☐☐	☐ 1	☐ 2	☐ 3	☐ 4

25 How many times ***in the last 3 months*** have you had:

Blood tests or other pathology	
X-rays or imaging *(eg CT scans)*	

SECTION 3: WORKING TOGETHER

This section asks how well you and your health care providers (eg GPs, specialists, practice nurses) work together to provide care for you.

If you see more than one health professional on an ongoing basis for a long-term illness, go to question 26 below.

If you do not see more than one health professional on an ongoing basis, skip to question 32.

26 **Who is the *main* person who organises your health care?** *Mark one only*

- ☐ 1 No one organises my health care
- ☐ 2 Specialist
- ☐ 3 Practice nurse
- ☐ 4 Myself
- ☐ 5 My partner, spouse
- ☐ 6 Relative
- ☐ 7 Friend
- ☐ 8 GP
- ☐ 9 Other *(Specify)*

27 ***In the last year*, when you visited a doctor or other professional, did they have information about you from other health professionals you had visited?**

- ☐ 1 Always
- ☐ 2 Usually
- ☐ 3 Sometimes
- ☐ 4 Never
- ☐ 5 Not applicable

28 **How did they get that information?**
Mark X for all that apply

- ☐ Information was sent to them from another health professional
- ☐ I took a letter to the appointment
- ☐ The information, test result or letter was already on their computer/desk
- ☐ I don't know
- ☐ Other *(Specify)*
- ☐ Not applicable

29 ***In the last year*, have you had to make another appointment because information was not available to them?**

☐ 1 Yes ☐ 2 No

30 ***In the last year*, have you had to have tests or imaging repeated because information was not available to them?**

☐ 1 Yes ☐ 2 No

31 ***In the last year*, have you had to have tests or imaging repeated because a doctor or other health professional wanted their own tests done?**

- ☐ 1 Yes
- ☐ 2 No
- ☐ 3 Don't know
- ☐ 4 Not applicable

32 **Has your GP or practice nurse given you a copy of a *written* management plan for your illness?**

- ☐ 1 Yes
- ☐ 2 No
- ☐ 3 Don't know
- ☐ 4 Not applicable

Black Harvest DTCH-2010_01 Chronic Health V5 06-12-10

33 Has any other health professional (eg specialist, nurse) given you a copy of a ***written*** management plan for your health conditions?

- ☐ 1 Yes
- ☐ 2 No
- ☐ 3 Don't know
- ☐ 4 Not applicable

34 Do you have your ***own written*** plan for managing your health that's different from questions 32 and 33 above?

- ☐ 1 Yes
- ☐ 2 No

35 How many ***prescribed*** medicines do you regularly take?

☐☐

If you do not take any prescribed medicines, skip to question 40.

36 In the last year, have there been occasions when your medicines were changed?

- ☐ 1 Yes ➔ **GO TO Q37.1**
- ☐ 2 No ➔ **GO TO Q39**

37.1 Think of the last time your prescribed medicines were changed; which of the following health professionals made that change?
Mark one only

- ☐ 1 GP
- ☐ 2 Specialist
- ☐ 3 Nurse
- ☐ 4 Other (Specify)

____

37.2 Did they explain to you why the change was made?

- ☐ 1 Yes
- ☐ 2 No

37.3 Did they ask if you were taking other medicines?

- ☐ 1 Yes
- ☐ 2 No

37.4 Did they explain what you needed to do about other medicines you were taking?

- ☐ 1 Yes
- ☐ 2 No
- ☐ 3 Not applicable

37.5 If your GP did not make the change, did they know about the change when you saw them next?

- ☐ 1 Yes
- ☐ 2 No
- ☐ 3 Don't know
- ☐ 4 Not applicable

37.6 Did a pharmacist explain the change to you?

- ☐ 1 Yes
- ☐ 2 No

38 Have you ever received conflicting information from different health professionals about the medicines you are taking?

- ☐ 1 Yes
- ☐ 2 No

39 In the last year, have you been to a hospital emergency department because your medicines caused a health problem?

- ☐ 1 Yes
- ☐ 2 No

40 How often do you have to change your other arrangements to fit in with your health appointments?

- ☐ 1 Often
- ☐ 2 Sometimes
- ☐ 3 Rarely
- ☐ 4 Never

Please check "go to"!!

12-10 Black Harvest DTCH-2010_01 Chronic Health V5 06-12-10

Please check "go to"!!

41 Do you have a GP or a practice you usually go to?

- ☐ 1 Yes ➔ GO TO Q42
- ☐ 2 No ➔ GO TO Q44

42 Why do you usually go to the same GP or practice? *Mark X for all that apply*

- ☐ The GP/practice bulk bills
- ☐ The GP/practice is close by
- ☐ Same cultural background or language
- ☐ Friendly GP/practice
- ☐ Takes a genuine interest
- ☐ I trust the GP/practice
- ☐ Availability of female doctors
- ☐ GP/practice provides a range of services
- ☐ Easier to get appointment with the same GP/practice
- ☐ Familiar with my case or history
- ☐ Has special interest in my condition
- ☐ After hours health care is available
- ☐ Other *(Specify)*

43 How long have you been going to this GP or practice? *Specify number of years **or** months*

Number of years: ☐☐

OR

Number of months: ☐☐

44 If you ***don't*** usually go to the same GP, why do you choose to go to different GPs or practices?

- ☐ Not applicable

45 If your GP refers you to other health professionals (eg specialist, dietician etc), does the GP follow up with you about what happened?

- ☐ 1 Always
- ☐ 2 Sometimes
- ☐ 3 Rarely
- ☐ 4 Never
- ☐ 5 Don't know
- ☐ 6 Not applicable

SECTION 4: TIME USE

These questions ask about how much time you spend on looking after your health.

46 ***On most days*** how much time do you generally spend on each of the following?

	Hours	Minutes
Sorting your medications		
Preparing your medications		
Taking your medications		
Carrying out treatments		
Testing or monitoring your health		
Preparing special foods		
Taking exercise/stretching		

47 ***In the last month*** **how much time did you spend on each of the following?**

	Hours	Minutes
Shopping for medicines, equipment or disposables, other necessary health items for yourself		
Shopping for special foods you may need for yourself		
Attending rehabilitation programs		
Attending health education or self-management programs		
Attending support groups, such as cancer or diabetes groups		
Looking for and reading health information		

48 ***In the last month*** **how much time did you spend on each of the following?**

	Hours	Minutes
Organising appointments for yourself		
Organising travel to and from health-related appointments		
Travelling to and from health-related appointments, including support groups		
Sitting in waiting rooms		
With the doctor or other health professional for consultation, advice or treatment		
Having blood tests, x-rays or other tests		
Having other medical treatments (eg dialysis, chemotherapy, radiotherapy)		

PART C — Carer's part

A **primary carer** is someone who provides most of the informal assistance to a person who needs help with daily life activities, or attends health care consultations or treatments with them.

If you are a primary carer, please answer the questions in this section.

If you are not a primary carer, you have finished the questionnaire. Thank you for taking the time to complete it. Please go to the back cover for instructions about returning the questionnaire.

49 **How many people do you care for?**

☐☐

50 **Do you live in the same residence as the *main* person you care for?**

☐ 1 Yes ☐ 2 No

51 **Is the main person you care for a member of your family?**

☐ 1 Yes ➔ GO TO Q52
☐ 2 No ➔ GO TO Q53

52 **Is this person your**

☐ 1 Parent
☐ 2 Partner/spouse
☐ 3 Son/daughter
☐ 4 Other

Please check "go to"!!

12-10 Black Harvest DTCH-2010_01 Chronic Health V5 06-12-10

53 Do you get paid for being a carer?

☐ 1 Yes ☐ 2 No

54 Do you keep a record of test results, medicines or treatment instructions for the main person you care for?

	Yes	No
A paper-based record	☐ 1	☐ 2
A computer-based record	☐ 1	☐ 2
A web-based record	☐ 1	☐ 2

55 Do you have a ***written*** plan to help manage the health of the main person you care for?

☐ 1 Yes ☐ 2 No

56 How often do you have to change your other arrangements to fit in with the health needs of the people you care for?

☐ 1 Often
☐ 2 Sometimes
☐ 3 Rarely
☐ 4 Never

57 How many of the people you care for have been told they have the following illnesses?

	Number of people you care for who have been told they have this illness
Cancer<br>*What type of cancer?* ☐	☐☐
Heart disease<br>*What type of heart disease?* ☐	☐☐
High blood pressure or Hypertension	☐☐
Stroke	☐☐
Diabetes	☐☐
Renal or kidney disease	☐☐
Asthma or Hay Fever	☐☐
Chronic Bronchitis or Emphysema or COPD<br>*(Chronic Obstructive Pulmonary Disease)*	☐☐
Arthritis	☐☐
Osteoporosis	☐☐
Chronic pain, including back pain	☐☐
Depression or Anxiety	☐☐
Other mental health condition	☐☐
Other illness lasting more than 6 months *(Specify)* ☐	☐☐
☐	☐☐

Black Harvest DTCH-2010_01 Chronic Health V5 06-12-10

The questions below ask about how much time you spend looking after the people you care for.

58 ***On most days*** **how much time do you generally spend on the following for all the people you care for?**

	Hours	Minutes
Sorting medications for all the people you care for		
Preparing medications for all the people you care for		
Giving medications to all the people you care for		
Carrying out treatments for all the people you care for		
Testing or monitoring the health for all the people you care for		
Preparing special foods for all the people you care for		
Looking for and reading health information for all the people you care for		

59 ***In the last month*** **how much time did you spend on the following for all the people you care for?**

	Hours	Minutes
Shopping for medicines, equipment or disposables, other necessary health items for all the people you care for		
Shopping for special foods you may need for all the people you care for		
Attending rehabilitation programs or health education sessions with the people you care for		
Attending health education sessions with the people you care for		
Attending support groups, such as cancer or diabetes groups, with the people you care for		

60 ***In the last month*** **how much time did you spend on the following for all the people you care for?**

	Hours	Minutes
Organising appointments for all the people you care for		
Organising travel to and from health-related appointments for all the people you care for		
Travelling to and from health-related appointments, including support groups		
Sitting in waiting rooms		
Being with them when they have consultations, advice or treatment with a doctor or other health professional		
Being with them when they have blood tests, x-rays or other tests		
Being with them when they have other medical treatments (eg dialysis, chemotherapy, radiotherapy)		

12-10 Black Harvest DTCH-2010_01 Chronic Health V5 06-12-10

61 How long did it take you to complete this questionnaire? [ ] minutes

62 Is there anything you would like to add about your health care, the health care system, or about about this study or questionnaire?

Thank you for completing this questionnaire.

Please return it using the addressed reply paid envelope provided.

This questionnaire is anonymous. If you are interested in participating in follow-up research some time in the future, please provide your contact details:

Name:

Address:

Town or Suburb: State or Territory: Postcode:

Home phone:

Mobile:

Email address:
(optional)

Barcode

NEED BARCODE????

Black Harvest DTCH-2010_01 Chronic Health V5 06-12-10
