# Supplementary material for: Time’s Up. Descriptive Epidemiology of Multi-Morbidity and Time Spent on Health Related Activity by Older Australians: A Time Use Survey
Source: PLoS One. 2013 Apr 1;8(4):e59379. doi: 10.1371/journal.pone.0059379 (PMC3613388; doi:10.1371/journal.pone.0059379)
Supplement: Attachment S2 — Response rates. (DOCX) [file pone.0059379.s002.docx]

**ATTACHMENT 2**

**RESPONSE RATES AND POPULATION COMPARISONS**

| ***National Diabetes Services Scheme*** | | |
| --- | --- | --- |
|  |  |  |
| Age | Gender | Response rate |
|  |  |  |
| 50-59 | Male | 11.5% |
|  | Female | 18.2% |
|  |  |  |
| 60-69 | Male | 17.3% |
|  | Female | 20.4% |
|  |  |  |
| 70-79 | Male | 18.7% |
|  | Female | 14.9% |
|  |  |  |
| 80-89 | Male | 17.5% |
|  | Female | 8.3% |
|  |  |  |
| All ages | Male | 16.7% |
|  | Female | 16.2% |
|  |  |  |
| Total | Total | 16.8% |
|  |  |  |
| ***Australian Lung Foundation*** | | |
|  |  |  |
| Gender | Response rate |  |
| Males | 23.7% |  |
| Females | 20.4% |  |
|  |  |  |
| Total | 22.0% |  |
|  |  |  |
| ***National Seniors Australia*** | |  |
|  |  |  |
| Age group | Response rate |  |
|  |  |  |
| 50-64 | 23.2% |  |
| 65-74 | 33.6% |  |
| 75 and over | 27.4% |  |
|  |  |  |
| Total | 28.4% |  |
|  |  |  |
