# Supplementary material for: Time’s Up. Descriptive Epidemiology of Multi-Morbidity and Time Spent on Health Related Activity by Older Australians: A Time Use Survey
Source: PLoS One. 2013 Apr 1;8(4):e59379. doi: 10.1371/journal.pone.0059379 (PMC3613388; doi:10.1371/journal.pone.0059379)
Supplement: File S1 — Tables (A–E). (DOCX) [file pone.0059379.s003.docx]

Table A: Socio-demographic and chronic disease characteristics of samples

*(Estimates weighted for non-response)*

|  | **Diabetes Sub-sample (N=427)** | **COPD Sub-sample (N=681)** | **NSA Sub-sample (N=1,432)** |
| --- | --- | --- | --- |
|  | *Percentage of sub-sample population* | | |
| *Gender* | | | |
| Male | 56.5 | 42.0 | 39.9 |
| Female | 43.5 | 58.0 | 60.1 |
|  |  |  |  |
| *Age* | | | |
| Less than 60 years | 25.0 | 11.3 | 26.9 |
| 60-69 years | 35.6 | 34.4 | 49.5 |
| 70-79 years | 24.8 | 37.5 | 15.3 |
| 80 years and over | 14.7 | 16.9 | 8.2 |
|  |  |  |  |
| *Region* | | | |
| Major Cities of Australia | 56.7 | 58.5 | 57.6 |
| Inner Regional Australia | 29.9 | 32.5 | 27.9 |
| Outer Regional Australia | 12.0 | 8.5 | 11.3 |
| Remote and Very Remote Australia | 1.4 | 0.5 | 3.2 |
|  |  |  |  |
| *Qualifications* | | | |
| No qualifications | 23.2 | 18.8 | 7.4 |
| Year 9 or year 10 schooling | 21.1 | 26.9 | 26.0 |
| Year 11 or year 12 schooling | 10.2 | 11.6 | 9.2 |
| Trade qualification | 13.5 | 11.3 | 7.2 |
| Certificate/diploma | 17.5 | 19.2 | 26.3 |
| Degree or higher degree | 14.5 | 12.3 | 23.9 |
|  |  |  |  |
| *Household Income (Australian dollars)* | | | |
| Less than $20,000 per year | 30.7 | 43.0 | 10.6 |
| $20-40,000 year | 32.2 | 34.5 | 27.4 |
| $40-60,000 year | 14.2 | 12.5 | 23.2 |
| $60-80,000 year | 6.8 | 5.1 | 13.7 |
| $80-100,000 year | 6.5 | 1.6 | 8.8 |
| $100,000 year or more | 9.5 | 3.3 | 16.3 |
|  | | | |
| *Number of chronic conditions ever diagnosed* | | | |
| Zero conditions | 1.4 | 1.0 | 15.9 |
| One conditions | 12.7 | 12.0 | 27.2 |
| Two conditions | 25.1 | 18.3 | 25.8 |
| Three conditions | 21.2 | 18.5 | 14.4 |
| Four conditions | 15.4 | 19.6 | 8.1 |
| Five or more conditions | 24.1 | 30.6 | 8.5 |
|  |  |  |  |
| *Number of medications regularly taken* | |  |  |
| No medication | 6.2 | 2.8 | 21.1 |
| One medication | 3.8 | 4.0 | 17.7 |
| Two medications | 6.7 | 5.8 | 17.8 |
| Three medications | 14.1 | 8.5 | 12.8 |
| Four medications | 16.7 | 12.9 | 10.1 |
| Five medications | 13.5 | 12.2 | 6.7 |
| Six or more medications | 39.1 | 53.9 | 14.0 |
|  |  |  |  |
| *Conditions ever diagnosed* |  |  |  |
| Cancer | 24.5 | 23.8 | 25.9 |
| Heart disease | 23.9 | 21.3 | 12.7 |
| Hypertension | 60.8 | 42.7 | 41.9 |
| Stroke | 8.9 | 6.0 | 3.0 |
| Diabetes Mellitus | 93.6 | 11.4 | 11.5 |
| Kidney disease | 8.0 | 4.9 | 3.5 |
| Asthma or hay fever | 18.5 | 37.4 | 19.1 |
| Chronic Obstructive Pulmonary Disease | 5.1 | 89.5 | 3.8 |
| Arthritis | 34.2 | 37.6 | 35.0 |
| Osteoporosis | 9.0 | 30.8 | 10.1 |
| Chronic pain, including back pain | 25.3 | 31.2 | 19.5 |
| Depression or anxiety | 20.4 | 28.0 | 17.4 |
| Other mental health condition | 5.0 | 2.6 | 1.4 |

Table B: Time spent on HRA across sub-samples

*(p values to test for trend using the Cuzick trend test)*

|  | | **Diabetes Sub-sample (N=427)** | **COPD Sub-sample (N=681)** | | | **NSA Sub-sample (N=1,432)** | |
| --- | --- | --- | --- | --- | --- | --- | --- |
|  | | | | | | | |
|  | | *Median Times reported in hours per month (95% CI)* | | | | | |
|  | | *Respondents who spent no time on HRA are included* | | | | | |
|  | | | | | | | |
| Total time | 11.1 (9.0-13.2) | | 16.5 (14.7-18.3) | | | 5.2 (4.7-5.6) |  |
|  | | | | | | | |
| *Gender* | |  |  | |  | | |
| Male | | 11.0 (8.1-13.9) | 17.7 (14.2-21.2) | | 4.8 (4.0-5.6) | | |
| Female | | 11.1 (7.5-14.7) | 15.9 (13.4-18.4) | | 5.3 (4.8-5.9) | | |
|  | |  |  | |  | | |
| *Age* | |  |  | |  | | |
| Less than 60 years | | 13.8 (9.1-18.4) | 28.0 (17.3-38.7) | | 5.0 (3.3-6.7) | | |
| 60-69 years | | 10.3 (7.0-13.6) | 16.3 (13.4-19.3) | | 4.5 (3.8-5.2) | | |
| 70-79 years | | 10.6 (6.7-14.5) | 17.5 (14.6-20.4) | | 5.3 (4.5-6.2) | | |
| 80 years and over | | 10.0 (4.2-15.8) | 11.9 (6.9-17.0) | | 8.7 (6.4-11.0) | | |
| *Test for trend (p-value )* | | *0.544* | *0.005* | | *<0.001* | | |
|  | |  |  | |  | | |
| *Region* | |  |  | |  | | |
| Major Cities of Australia | | 10.5 (7.8-13.2) | 16.5 (14.0-19.0) | | 5.4 (4.8-6.0) | | |
| Inner Regional Australia | | 12.5 (9.6-15.4) | 17.6 (14.1-21.1) | | 4.8 (3.9-5.8) | | |
| Outer Regional Australia | | 15.0 (8.1-21.9) | 17.5 (10.6-24.4 | | 3.7 (1.5-5.8) | | |
| Remote and Very Remote Australia | | NA | NA | | 5.2 (0.5-9.9) | | |
| *Test for trend (p-value )* | | *0.711* | *0.984* | | *0.473* | | |
|  | |  |  | |  | | |
| *Qualifications* | |  |  | |  | | |
| No qualifications | | 7.6 (3.0-12.2) | 13.5 (9.3-17.7) | | 4.5 (1.4-7.6) | | |
| Year 9 or year 10 schooling | | 10.3 (6.1-14.4) | 16.5 (12.0-21.0) | | 5.1 (4.1-6.1) | | |
| Year 11 or year 12 schooling | | 13.6 (7.7-19.5) | 15.0 (9.6-21.4) | | 6.0 (3.9-8.1) | | |
| Trade qualification | | 10.5 (5.7-15.3) | 23.0 (17.6-28.4) | | 4.3 (1.8-6.7) | | |
| Certificate/diploma | | 16.3 (11.7-21.0) | 16.0 (12.7-19.3) | | 5.7 (4.7-6.6) | | |
| Degree or higher degree | | 18.5 (9.3-27.7) | 21.3 (12.45-30.0) | | 4.3 (2.9-5.7) | | |
| *Test for trend (p-value )* | | *0.011* | *0.025* | | *0.678* | | |
|  | |  |  | |  | | |
| *Household Income (Australian dollars)* | |  |  | |  | | |
| Less than $20,000 per year | | 10.3 (6.8-13.8) | 18.3 (15.5-21.1) | | 8.5 (6.2-10.8) | | |
| $20-40,000 year | | 10.5 (6.2-14.8) | 15.6 (11.4-19.8) | | 6.0 (4.8-7.2) | | |
| $40-60,000 year | | 12.5 (7.0-18.0) | 18.0 (13.5-22.5) | | 4.0 (2.7-5.3) | | |
| $60-80,000 year | | 9.0 (3.3-14.7) | 12.3 (5.2-19.5) | | 4.75 (2.9-6.6) | | |
| $80-100,000 year | | 10.8 (0.5-21.0) | NA | | 2.8 (1.5-4.2) | | |
| $100,000 year or more | | 15.0 (6.2-23.8) | 19.5 (0-41.4) | | 5.0 (4.0-6.0) | | |
| *Test for trend (p-value )* | | *0.332* | | *0.439* | *<0.001* | | |
|  | | | | | | | |
| *Number of chronic conditions ever diagnosed* | | | | | | | |
| Zero conditions | | NA** | NA** | | | 1.4 (0.8-2.0) | |
| One conditions | | 5.8 (0.7-10.8) | 13.3 (8.6-18.1) | | | 3.0 (2.7-3.3) | |
| Two conditions | | 6.6 (4.3-8.9) | 12.8 (7.5-18.0) | | | 4.9 (4.3-5.5) | |
| Three conditions | | 11.4 (9.2-13.7) | 13.8 (10.6-17.1) | | | 10.2 (8.9-11.4) | |
| Four conditions | | 16.0 (9.5-22.5) | 15.7 (12.0-19.3) | | | 9.5 (6.6-12.4) | |
| Five or more conditions | | 16.5 (9.9-23.1) | 26.7 (20.4-32.9) | | | 21.5(17.5-25.5) | |
| *Test for trend (p-value )* | | *<0.001* | *<0.001* | | | *<0.001* | |
|  | |  |  | | |  | |
| *Number of medications taken regularly* | | |  | | |  | |
| No medication | | 0.5 (0-8.0) | 3.2 (0-11.0) | | | 1.0 (0.7-1.3) | |
| One medication | | 4.1 (0-10.1) | 8.5 (2.9-14.1) | | | 3.3 (2.7-4.0) | |
| Two medications | | 4.4 (2.5-6.4) | 5.0 (0.5-9.5) | | | 4.8 (3.7-5.8) | |
| Three medications | | 9.5 (1.7-17.3) | 11.5 (4.8-18.3) | | | 5.9 (4.8-7.0) | |
| Four medications | | 8.1 (3.8-12.4) | 11.5 (6.8-16.2) | | | 7.8 (6.2-9.5) | |
| Five medications | | 12.6 (9.1-16.1) | 14.7 (8.7-20.7) | | | 10.8 (9.6-11.9) | |
| Six or more medications | | 15.6 (12.0-19.2) | 21.0 (18.5-23.5) | | | 18.8 (15.3-22.3) | |
| *Test for trend (p-value )* | | *<0.001* | *<0.001* | | | *<0.001* | |
|  | |  |  | | |  | |
| *Conditions ever diagnosed* | |  |  | | |  | |
| Cancer | | 17.0 (11.1-22.9) | 17.2 (13.1-21.2) | | | 7.6 (6.0-9.2) | |
| Heart disease | | 15.0 (10.3-19.7) | 19.5 (13.3-25.7) | | | 10.8 (9.8-11.8) | |
| Hypertension | | 12.3 (10.2-14.3) | 17.6 (14.1-21.1) | | | 7.7 (6.8-8.6) | |
| Stroke | | 15.6 (8.7-22.4) | 21.0 (11.5-30.5) | | | 8.6 (4.7-12.5) | |
| Diabetes Mellitus | | 11.4 (9.2-13.6) | 32.8 (22.9-42.8) | | | 12.2 (9.9-14.5) | |
| Kidney disease | | 19.7 (3.3-36.1) | 34.7 (21.6-47.8) | | | 12.0 (2.3-21.7) | |
| Asthma or hay fever | | 10.3 (4.9-15.6) | 22.4 (19.1-25.7) | | | 8.8 (7.1-10.6) | |
| Chronic Obstructive Pulmonary Disease | | 19.9 (8.8-30.9) | 17.4 (15.3-19.5) | | | 20.3 (15.8-24.7) | |
| Arthritis | | 14.3 (11.7-16.9) | 17.5 (14.9-20.1) | | | 7.8 (6.7-8.8) | |
| Osteoporosis | | 22.8 (11.5-34.0) | 19.5 (16.0-23.0) | | | 10.5 (8.3-12.7) | |
| Chronic pain, including back pain | | 13.8 (9.6-17.9) | 19.5 (14.8-24.2) | | | 11.7 (9.9-22.7) | |
| Depression or anxiety | | 16.3 (10.0-22.7) | 23.0 (16.1-29.9) | | | 10.8 (7.8-13.9) | |
| Other mental health condition | | 17.5 (0-38.4) | 18.9 (5.8-32.0) | | | 28.5 (3.9-53.1) | |

i) National Seniors Australia n = 1432

ii) Australian Lung Foundation n= 681

iii) National Diabetes Services Scheme n= 427

*Median hours per month (CI 95%)

**Not available as cell size had less than 10 observations

Table C: Structure of time use: reported activities and median time spent on each

|  | **Diabetes Sub-sample** | **COPD Sub-sample** | **NSA Sub-sample** | |
| --- | --- | --- | --- | --- |
|  | **Percentage reporting the activity** | | | |
|  | *(Estimates and 95% CI)* | | | |
| Home activities excluding exercise | 91.2 (88.3-94.1) | 94.5 (92.7-96.3) | 76.4 (74.0-78.7) | |
| Home activities including exercise | 93.2 (91.2-95.4) | 96.2 (94.8-97.7) | 92.4 (90.9-93.8) | |
| Clinic activities | 82.4 (78.3-86.6) | 87.8 (85.4-90.3) | 76.7(74.8-78.7) | |
| Other activities | 80.8 (76.9-84.8) | 84.6 (81.5-87.7) | 75.2 (73.3-77.1) | |
| Total time excluding exercise | 95.1 (93.1-97.1) | 97.8 (96.7-98.8) | 92.6 (91.4-93.8) | |
| Total time including exercise | 95.5 (94.0-97.1) | 98.3 (97.4-99.2) | 96.4 (95.4-97.4) | |
|  | **Median Time spent by those spending time on these activities**  **(hours per month )** | | | |
|  | *(Estimates and 95% CI)* | | | |
|  | *(ie. Excluding those with zero hours)* | | | |
| Home activities excluding exercise | 7.5 (6.3-8.7) | 7.5 (6.7-8.3) | 3.0 (2.5-3.5) | |
| Home activities including exercise | 20.5 (17.6-23.6) | 22.5 (21.1-23.9) | 18.0 (17.1-18.9) | |
| Clinic activities | 2.2 (1.9-2.5) | 4.0 (3.3-4.6) | 2.4 (2.2-2.6) | |
| Other activities | 1.0 (0.76-1.24) | 2.0 (1.6-2.4) | 0.7 (0.5-0.8) | |
| Total time excluding exercise | 12.25 (10.0-14.5) | 17.5(15.5-19.5) | 6.0 (5.6-6.4) | |
| Total time including exercise | 26.2 (22.6-29.7) | 31.5 (29.5-33.5) | 23.0 (21.3-24.7) | |
|  | **Median Times reported in hours per month across all respondents** | | | |
|  | *(Estimates and 95% CI)* | | | |
|  | *(ie. Including those with zero hours)* | | | |
| Home activities excluding exercise | 6.0 (4.6-7.4) | 7.5 (6.8-8.2) | | 1.5 (1.0-2.0) |
| Home activities including exercise | 19.0 (16.2-21.8) | 21.0 (19.3-22.7) | | 16.5 (15.9-17.1) |
| Clinical visits | 1.7 (1.4-1.9) | 3.0 2.5-3.5) | | 1.5 (1.3-1.7) |
| Other activities | 0.5 (0.4-0.6) | 1.5 (1.2-1.8) | | 0.3 (0.3-0.4) |
| Total time excluding exercise | 11.1 (9.0-13.2) | 16.5 (14.7-18.3) | | 5.2 (4.7-5.6) |
| Total time including exercise | 25.8 (22.0-29.5) | 31.2 (29.1-33.2) | | 21.7 (20.3-23.0) |

*Note: These numbers do not add to the totals as each percentage or median is based on a different group of people who have provided valid responses.*

Table D: Distribution of Total time use by sample component

| Total time use (hours per month) | **Diabetes Sub-sample** | **COPD Sub-sample** | **NSA Sub-sample** |
| --- | --- | --- | --- |
|  | *Percentage of sub-sample population* | | |
| Nil | 4.9 | 2.1 | 7.4 |
| 20 Hours or less | 64.1 | 53.3 | 73.2 |
| 20-40 hours | 16.0 | 25.9 | 11.3 |
| 40-60 Hours | 7.4 | 8.1 | 3.6 |
| 60-80 Hours | 2.7 | 3.6 | 1.9 |
| 80-100 Hours | 2.8 | 1.3 | 0.7 |
| 100 Hours or more | 2.1 | 5.6 | 1.9 |
|  |  |  |  |
| Total | 100.0 | 100.0 | 100.0 |

Table E: Total time use by top 10% in each category by number of chronic illnesses and sample component

*(The top 10% in each category use more time each month than the 90^th^ percentiles reported below)*

| Number of chronic illnesses | | **Diabetes Sub-sample** | | **COPD Sub-sample** | | **NSA Sub-sample** |
| --- | --- | --- | --- | --- | --- | --- |
|  | | 90th percentile: hours per month (95% CI) | | | | |
| 0 | | NA | | NA | | 19.3(8.7-29.8) |
| 1 | | 26.2(16.0-36.4) | | 40.0(11.0-69.0) | | 23.0(15.4-30.6) |
| 2 | | 36.1(20.8-51.4) | | 43.5(34.2-57.8) | | 31.0(23.4-38.6) |
| 3 | | 41.5(28.9-54.1) | | 44.0(33.7-54.3) | | 37.5(30.6-44.4) |
| 4 | | 83.0(45.0-121.0) | | 56.3(41.0-71.6) | | 44.5(27.1-61.9) |
| 5 or more | | 80.1(60.2-100.0) | | 109.5(85.7-133.3) | | 71.5(34.0-109.0) |
|  | | | | | | |
| Total | 51.4(43.0-59.8) | | 62.6(53.5-71.6) | | 34.1(30.7-37.5) | |
